# Integration of human papillomavirus 16 in esophageal carcinoma samples

**DOI:** 10.1186/s13027-017-0164-3

**Published:** 2017-10-13

**Authors:** Shuying Li, Haie Shen, Zhanjun Liu, Ning Li, Suxian Yang, Ke Zhang, Jintao Li

**Affiliations:** 1 North China University of Science and Technology (Hebei Key Laboratory for Chronic Diseases, Tangshan Key Laboratory for Preclinical and Basic Research on Chronic Diseases), No.21 Bohai Road, Caofeidian New Town, Tangshan City, Hebei Province 063210 People’s Republic of China; 2 College of Life Science and Bio-engineering, Beijing University of Technology, Beijing, city, 100124 People’s Republic of China

**Keywords:** Esophageal carcinoma, Human papillomavirus, Infection, Integration, Etiology

## Abstract

**Background:**

Esophageal carcinoma (EC) is one of the major cancers in China. In 1982, Syrjanen first hypothesized the relationship between human papillomavirus (HPV) infection and the development of esophageal cancer. Since then, many reports in the field have supported this viewpoint. This study investigated the etiological relationship between HPV infection and the occurrence of esophageal carcinoma at Tangshan City of the Hebei province in China.

**Methods:**

189 samples of esophageal carcinoma patients were collected. DNA and RNA were isolated from samples, HPV DNA was detected by polymerase chain reaction (PCR) using My09/11 for HPV L1, and HPV16 was determined using type-specific primer sets for HPV16 E6. The HPV16 integration site was verified by amplification of papillomavirus oncogene transcripts, and HPV16 oncogene transcript products were ligated to the pMD-18 T vector and sequenced to confirm the physical location of HPV16 integration.

**Results:**

168 HPV-positive samples were detected in 189 samples, and among them 76 specimens were HPV16 positive. Approximately 600 bp of the HPV16 oncogene transcript were detected in nine esophageal cancer samples. Sequence analysis revealed that HPV16 E7 integrated into human chromosome 2 in three samples, into human chromosome 5 in one sample, into human chromosome 6 in one sample, into human chromosome 8 in two samples, and into human chromosome 17 in two samples. The results verified that the integrated HPV16 E7 in five samples harbored one mutation of viral DNA compared with the HPV16 sequence provided in GenBank (K02718).

**Conclusions:**

The high prevalence of HPV16 suggests that HPV16 may play an etiological role in the development of esophageal cancer. The integration of HPV16 into host cell chromosomes suggests that persistent HPV infection is key for esophageal epithelial cell malignant transformation and carcinogenesis.

## Background

Esophageal carcinoma (EC) is one of the major cancers in China [1, 2]. Environmental factors and life styles of esophageal carcinoma patients have been widely researched [3–5], although the pathogeny of esophageal cancer has not yet been determined. In 1982, Syrjanen first hypothesized the relationship between human papillomaviruses (HPV) infection and the development of esophageal cancer [6]. Since then, many reports in the field have supported this viewpoint [7–13]. Our previous work showed that high-risk HPV types 16 and 18 were detected in esophageal tumors [14], and HPV18 was localized in human chromosome 8 in the EC109 cell line [15], these results indicates that HPV infection is a pathogenic factor for esophageal cancer.

Few studies have described the HPV integration site, so the objective of the current work to discuss HPV16 infection and integration site in the human genome to better understand its role in esophageal cancer. HPV infection detected using My09/11 for HPV L1 (16), HPV16 was determined using type-specific primer sets for HPV16 E6, and integration site of HPV16 in esophageal cancer was analyzed by amplification of papillomavirus oncogene transcripts (APOT), which allowed the discrimination of HPV mRNAs derived from integrated genomes [17]. The integration of HPV in the host chromosome integration site can be accurately located by detection of the transcription of poly (A) tail [18, 19]. Namely, first, cDNA was synthesized by reverse transcription using RNA as template, and (dT)_17_-p3 as primer; second, PCR amplification was conducted using cDNA as template, p1-HPV16 E7 and p3 as primers; third, PCR was conducted using above PCR product as template, p2-HPV16 E7 and (dT)_17_-p3 as primers; fourth, the PCR product was cloned into a pMD-18 T vector; fifth, sequencing analysis and blast in GenBank. The integration sites was determined by sequence alignment including both HPV and human chromosome sequence.

## Materials

### Sample collection and preparation

A total of 189 fresh surgically resected tissue samples and clinical information of patients were obtained in 2013.03 to 2015.12 after participants authorized and signed informed consent forms to participate in the study. All specimen donors were pathologically diagnosed with esophageal carcinoma, and treated at the pathology department of Tangshan people’s hospital in Hebei province. The patients were from the Tangshan area, 136 cases were male, and 53 cases were female. The average age of subiects was 58 (range 40–76) years old. Subject were classified as follows according to clinical and pathological stages of esophagus carcinoma: 98 early-stage;, 63 middle -stage; and 28 late-stage. Tumor tissue differentiation was separated into 30 well-differentiated types, 104 moderately-differentiated types and 55 poorly-differentiated types. All fresh samples were stored at −80 °C prior to experiments.

HPV16/pBR322 and HPV18/pBR322 DNA plasmid containing the whole genome of HPV16 and HPV18, plasmid of beta-actin DNA containing part human housekeeping genes, and human embryonic kidney 293 (HEK293) cell line DNA were stored at −20 °C prior to experiments.

## Methods

### DNA extraction

DNA was extracted from each tissue specimen (approximately 25 mg) using a QIAamp DNA mini kit (QIAGEN, Hilden, Germany) according to the manufacturer’s instructions, and each sample DNA was eluted with approximately 50 μl sterilized distilled water. The concentration of each extracted DNA was detected and diluted to 100 ng/μl. DNA was stored at −20 °C.

### Detection of specimen quality and HPV DNA

The quality of each tissue sample DNA was analyzed by PCR amplification using housekeeping gene β-actin primers [20], HEK293 cell line DNA was used as a positive control, sterile water was used as a negative control to ensure the quality of specimens.

HPV DNA of each specimen was detected by PCR amplification using My09/11 primers for HPV L1 (My09: 5′-CGTCCMARRGGAWACTGATC-3′, MY11: 5′-GCMCAGGGWCATAAYAATGG-3′, PCR products 450 bp) [16] and HPV16 E6-specific primers (forward: 5′- ACTGCGACGTGAGGTATATGAC-3′, reverse: 5′- TTGATGATCTGCAACAAGACATAC-3′, PCR products 320 bp), which were designed according to the GenBank-provided HPV16 gene sequences K02718.1 (http://www.ncbi.nlm.nih.gov/nuccore/K02718). Primers were synthezed by Sangon Biotech of Shanghai. The PCR products were resolved on a 1.0% agarose gel with Goldview I nuclear staining dye (BioTeke Corporation, Beijing, China) and observed with a UV transilluminator.

Total PCR reaction was performed using Ex Taq Polymerase kit (Takara Biotechnology Co., Ltd., Dalian, China) in a 25-μl volume containing 5 pmol each of the forward and reverse primers, 1 × Ex Buffer (MgCl_2_ free), 0.2 mM mixture deoxynucleoside triphosphate (dNTPs), 2.5 mM MgCl_2_, 1 U Ex Taq DNA polymerase, and 0.25 ng extracted DNA template and control template were added to the reaction system. The following cycling was used: 95 °C for 5 min, followed by 31 cycles of 95 °C for 30 s, 55 °C for 30 s, and 72 °C for 30 s. The final extension step was 72 °C for 5 min, and stored at 4 °C.

### Reverse transcription (RT)

Every sample RNA was extracted using the RNeasy Mini kit (Qiagen GmbH, Hilden, Germany) according to manufacturer’s instruction. Reverse transcription (RT) was performed using M-MLV Reverse Transcriptase kit (BioTeke Corporation, Beijing, China) according to the manufacturer’s protocol in a total volume of 20 μl, consisting of 9.2 μl RNase-free H2O, 4 μl of 5× first-strand buffer, 1.0 μl RNase inhibitor (40 U), 50 ng total RNA, 1 μl (dT)17-p3 (10 pmol primer: GACTCGAGTCGACATCGATTTTTTTTTTTTTTTTT), 0.5 μl dNTPs (0.2 mM), and 1 μl super script reverse transcriptase (200 U). The RNA reverse transcription was performed at 42 °C for 50 min and deactivated at 70 °C for 15 min, and the resulting product was stored at 4 °C.

To ensure mRNA quality of each tissue sample, human housekeeping gene GAPDH was detected using RT-PCR for verification of viral-cell fusion transcripts. The RT reactions were performed according to above, and the cDNA was stored at 4 °C. The PCR was conducted using a Takara Ex Taq Polymerase kit in a 20 μl reaction mixture containing 1X Ex buffer (MgCl_2_-free), 0.2 mM dNTPs, 2.5 mM MgCl_2_, 1 unit Ex Taq DNA polymerase, 5 pmol each GAPDH primer (forward, 5′-CATCACCATCTTCCAGGA-3′ and reverse, 5′-GTCTACCACCCTATTGCA-3′) and 2 μl cDNA template. The PCR cycling profile was as follows: 95 °C for 5 min; followed by 31 cycles of 95 °C for 30 s, 52 °C for 30 s and 72 °C for 30 s; followed by a final extension at 72 °C for 5 min; and storage at 4 °C.

### The viral-cell fusion transcripts analysis

The first PCR amplification was conducted according to Klaes et al. [17] in a 20 μl volume using HPV16 E7-specific forward primers for p1–16 (5′-CGGACAGAGCCCATTACAAT-3′) and reverse primers for p3 (5′-GACTCGAGTCGACATCG-3′); the reaction system included 1 × Ex buffer, 2.5 mM MgCl_2_, 0.2 mM dNTPs, 5 pM primers, 2 μl cDNA, and 1 U Ex Taq DNA polymerase. The PCR cycle was as follows: 95 °C for 5 min, followed by 30 cycles at 95 °C for 1 min, 56 °C for 1 min, 72 °C for 3 min, and a final extension at 72 °C for 5 min, and stored at 4 °C.

Nested PCR was performed with identical conditions except for the annealing temperature at 67 °C with the following primers: HPV16 E7-specific forward primer p2–16 (5′-CTTTTTGTTGCAAGTGTGACTCTACG-3′) and reverse primer (dT)17-p3; 5 μl of the first round PCR product was used as template. To ensure specificity of these primers, HEK293 cell line DNA template was used as a negative control. The PCR products were resolved on a 1.0% agarose gel with Goldview I nuclear staining dye (BioTeke Corporation, Beijing, China) and observed with a UV transilluminator.

### Cloning and sequence analysis for HPV16 integrated position in the human chromosome

The final nested PCR products were cloned into pMD-18 T vector according to reference [21], except the temperature of the target segment ligation to the pMD-18 T vector was changed to room temperature for 1 h. Then, commissioning Beijing Rui Bo Xing ke Biological Technology company to sequence. The sequencing results were blasted at NCBI (https://blast.ncbi.nlm.nih.gov/Blast.cgi?PROGRAM=blastn&PAGE_TYPE=BlastSearch&LINK_LOC=blasthome), and HPV16 integration sites were determined in human chromosome for every integration specimen, severally.

### Statistical analysis

Statistical analysis was performed using Statistical Package for the Social Sciences (SPSS) version 13.0 software. *P* –value of less than 0.05 was considered as statistically significant.

## Results

### Detection of specimen quality and HPV DNA

290-bp PCR products of β-actin were detected in 189 DNA samples, this result indicated that these DNA samples were in high quality and could meet the requirements for further experiments.

**Fig. 1a:**
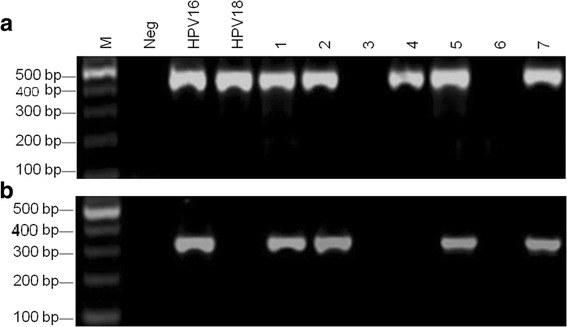
Detection of HPV DNA using MY09/11 primers in esophageal carcinoma samples. M was 100 bp DNA ladder; Neg was a negative control; lanes 1–7 were detection of HPV DNA in different esophageal carcinoma samples. HPV16 and 18 were positive controls with the HPV16/pBR322 and HPV18/pBR322 templates. **b** Detection of HPV16 DNA using HPV16 E6 specific primers in esophageal carcinoma samples. M was 100 bp DNA ladder; Neg was a negative control; lanes 1–7 were detection of HPV16 DNA in different esophageal carcinoma samples. HPV16 was a positive control with HPV16/pBR322 template; HPV18 was a specific control using HPV18/pBR322

168 specimens were detected HPV positive among 189 samples using MY09 / 11 primers (Fig. 1a and b). All HPV-positive samples were amplified using HPV16 E6 specific primer sets, and among them 76 specimens were HPV16 positive (Fig. 1a and b).

### HPV16 integration derived transcript in HPV16 E6 positive esophageal cancer samples

**Fig. 2:**
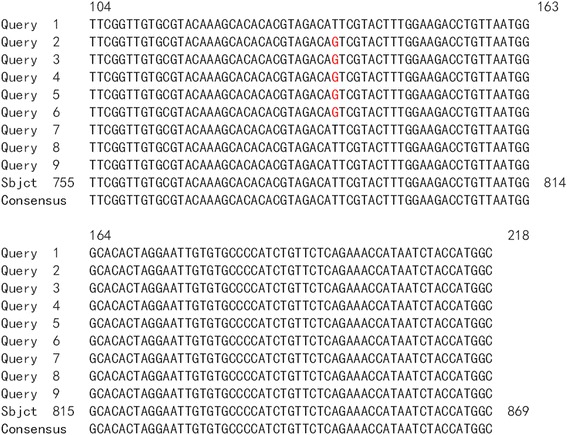
Alignment sequencing results compared with HPV16. Query 1–9: sequencing results for nine esophageal carcinoma specimens after PCR amplification with P2–16 E7-specific primers; Sbjct: part sequence of HPV 16E7-E1 in GenBank (K02718)

**Fig. 3:**
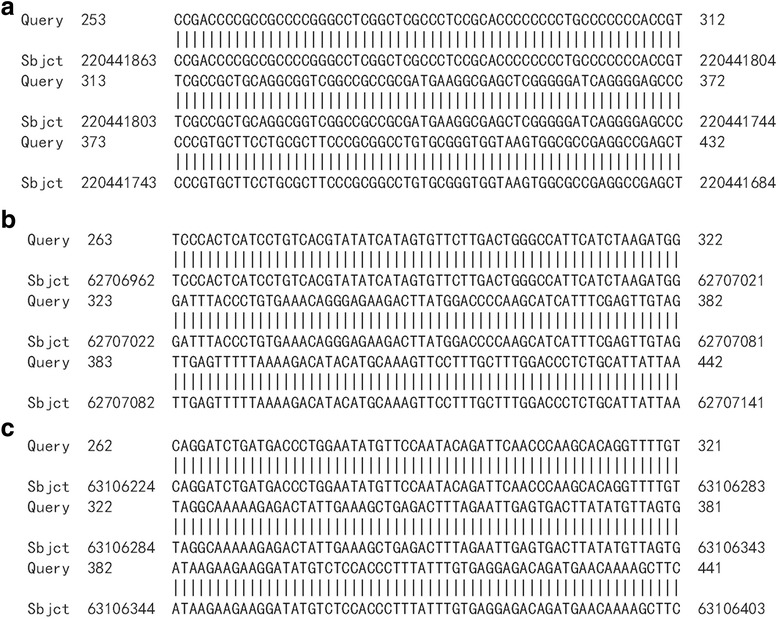
Alignment sequencing results compared with human chromosome 2. **a-c**: Alignment sequencing results of three esophageal carcinoma specimens compared with human chromosome. Query: sequencing results for three esophageal carcinoma specimens after PCR amplification with P2–16 E7-specific primers; Sbjct: part sequence of human chromosome 2

**Fig. 4:**
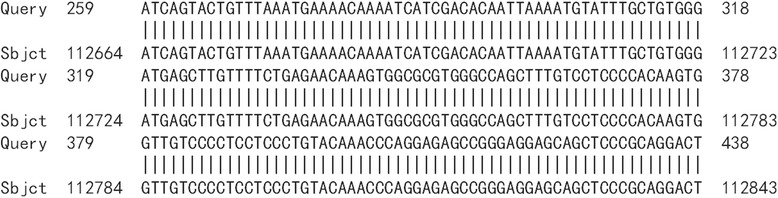
Alignment sequencing results compared with human chromosome 5. Alignment sequencing results of one esophageal carcinoma specimens compared with human chromosome. Query: sequencing results for one esophageal carcinoma specimens after PCR amplification with P2–16 E7-specific primers; Sbjct: part sequence of human chromosome 5

**Fig. 5:**
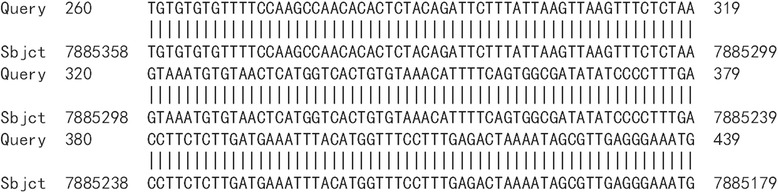
Alignment sequencing results compared with human chromosome 6. Alignment sequencing results of one esophageal carcinoma specimens compared with human chromosome. Query: sequencing results for one esophageal carcinoma specimens after PCR amplification with P2–16 E7-specific primers; Sbjct: part sequence of human chromosome 6

**Fig. 6:**
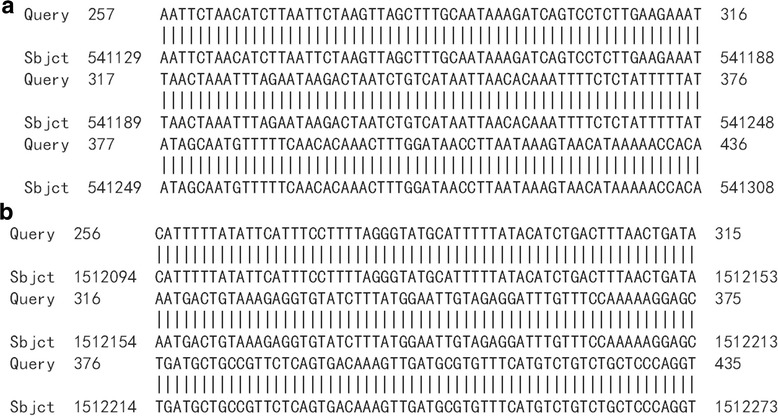
Alignment sequencing results compared with human chromosome 8. **a**, **b**: Alignment sequencing results of two esophageal carcinoma specimens compared with human chromosome. Query: sequencing results for two esophageal carcinoma specimens after PCR amplification with P2–16 E7-specific primers; Sbjct: part sequence of human chromosome 8

**Fig. 7:**
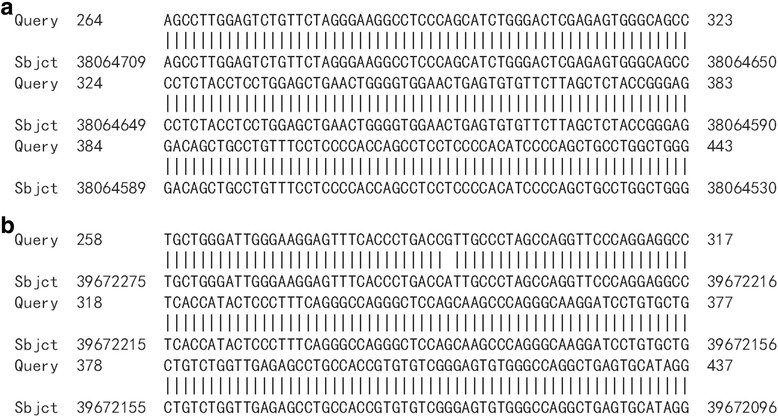
Alignment sequencing results compared with human chromosome 17. **a,b**: Alignment sequencing results of two esophageal carcinoma specimens compared with human chromosome. Query: sequencing results for two esophageal carcinoma specimens after PCR amplification with P2–16 E7-specific primers; Sbjct: part sequence of human chromosome 17

HPV16 integrated positions were confirmed in the HPV16 positive samples. Approximately 600 bp PCR products were detected from nine HPV16 E6 positive samples by APOT. HPV16 E7 PCR products in nine samples were ligated into the pMD-18 T vector, and sequence analysis of HPV16 integration sites was performed. The sequence analysis showed that HPV16 E7 PCR products of nine samples were part of the HPV16 E7-E1 sequence, and compared with the HPV16 sequence provided in GenBank (K02718), the analysis results verified that integrated HPV16 E7 harbored one mutation from five samples of viral DNA (Fig. 2). Partial sequences from nine samples were similar to human chromosome sequences, as follows: three were similar to human chromosome 2 (Fig. 3a, b and c). One was similar to human chromosome 5 (Fig. 4), one was similar to human chromosome 6 (Fig. 5), two were similar to human chromosome 8 (Fig. 6a and b), and two were similar to human chromosome 17 (Fig. 7a and b).

### The relationship between HPV16 integration and patient background

A total of fresh surgically resected tissue samples from 189 patients who were pathologically diagnosed with esophageal carcinoma were evaluated. The relationship between HPV16 integration and the patients’ backgrounds are shown in Table 1. Statistical analysis showed that HPV16 integration was not significantly correlated with gender, age, histological differentiation and pathological stage.

**Table 1:** Background of esophageal cancer patients and HPV16 integration

Background	HPV16 positive(*n* = 79)	Samples (*n* = 189)	Integrated	Without	*X* ^2^	*P* ^a^
				integrated		
sex					0.06	>0.05
male	55	136	7	48		
female	21	53	2	19		
age (year old)					1.38	>0.05
≤ 45	18	41	1	17		
46–64	28	70	3	25		
≥ 65	30	78	5	25		
differentiated type					0.09	>0.05
well differentiated	11	30	1	10		
moderately differentiated	49	104	6	43		
poorly differentiated	16	55	2	14		
pathological stages					2.99	>0.05
early stage	25	98	2	23		
middle stage	40	63	4	36		
late stage	11	28	3	8		
Total	76	189	9	67		

^a^Notice: There were no statistically significant difference (*P*>0.05)

## Discussion

High-risk HPV infection (such as HPV types16, HPV18, HPV31, HPV33, HPV35, HPV39, HPV45, HPV51, HPV52, HPV56, HPV58 and HPV59) have been identified as causative agents in cervix cancers [22, 23]. However, HPV has not been determined as to be a pathogenic factor for esophageal cancer occurrence thus far in highly prevalent regions [24, 25].

In the present study, HPV DNA from 189 patient tissue samples with pathologic diagnosis of esophageal carcinoma were examined, and a high prevalence was found (approximately 89% HPV DNA positive rate), and the HPV16 positive rate was 40.2%; Mehryar et al. [26] study showed that the prevalence of HPV types 16 and 18 was 40.40% and 47.47% in esophageal carcinoma for Tangshan, Hebei province, China, respectively, Dong et al. [7] study showed that Six HPV genotypes (HPV6, HPV16, HPV33, HPV39, HPV51, and HPV82) were present in at least 51.7% of the esophagealcarcinoma tissues, and combined with other studies [8–11], these findings indicate that HPV infection may be a pathogenic factor for esophageal cancers.

HPV16 integration was discovered in esophageal cancer cells from nine patient specimens, and HPV16 was found to be integrated into chromosomes 2, 5, 6, 8 and 17. With the integration of HPV18 in EC109 cells, these results indicate that HPV randomly integrates into the host chromosome and that the HPV viral genome is cleaved at the E1 and E2 ORFs for integration. Moreover, the E2 gene serves as a pivotal modulator for E6 and E7 gene expression in the viral life cycle. In many HPV-infected patients, HPV E2 restrains E6 and E7 gene transcription, which aids in the regulation of cellular proliferation [27]. Cleaving the E2 gene increased HPV E6 and E7 gene expression, which disrupts the cell cycle and leads to aberrant proliferation [28–30]. HPV E6 and E7 genes usually integrate into the host cell genome and require longer incubation periods for viral DNA replication and recombination to produce a variety of genetic changes in the viral and human genome. The expression levels of E6 and E7 simultaneously increased, which resulted in human chromosomal instability and the development of malignant tumors [18]. On the other hand, among nine integrated HPV16 specimens, three patient samples were in the late stage, including two females and one male, four patient samples from males were in the middle stage, and two patient samples from males were in the early stage; one was a well-differentiated sample, and six were moderately-differentiated samples, and two were poorly differentiated samples. Nine patient samples seem too small to be able to indicate that HPV16 integration is related to patient background (including gender, age, degree of differentiation, and pathological stages) and supports the assumption of preferred selective outgrowth of HPV-infected cells in preneoplastic lesions that express integrated viral oncogenes E6 and E7. The sequencing results showed one mutation for five DNA samples compared with the GenBank-provided HPV16 gene sequences of K02718.1 (http://www.ncbi.nlm.nih.gov/nuccore/K02718). This phenomenon may be different for local epidemic strains of HPV16. Next we will be to detect other HPV types infection and integration sites.

## Conclusions

In this study, 76 specimens were HPV16 positive in 189 esophageal carcinoma samples, this result suggested that a high prevalence of HPV16 plays an etiological role in the development of esophageal cancer. The integration of HPV16 into host cell chromosomes suggests that persistent HPV infection is key for esophageal epithelial cell malignant transformation and carcinogenesis. However, HPV infection may be one of multiple risk factors of esophageal cancer. Further work is needed to elucidate the underlying mechanism, other types of HPV integration sites, the genetic changes associated with HPV infection, and the molecular mechanism of esophageal cancer occurrence.

## Abbreviations

APOT: Amplification of papillomavirus oncogene transcripts.
EC: Esophageal carcinoma
HPV: Human papillomavirus
PCR: Polymerase chain reaction

## References

[1] He L, Fan J-H, Qiao Y-L (2017). Epidemiology, etiology, and prevention of esophageal squamous cell carcinoma in China. Cancer Biol Med.

[2] Zhao J, He YT, Zheng RS, Zhang SW, Chen WQ: Analysis of esophageal cancer time trends in China, 1989–2008. Asian Pac J Cancer Prev 13:4613-4617, 2012. 10.7314/apjcp.2012.13.9.4613 23167389

[3] Sun X, Chen W, Chen Z, Wen D, Zhao D, He Y (2010). Population-based casecontrol study on risk factors for esophageal cancer in five high-risk areas in China. Asian Pac J Cancer Prev.

[4] Gholipour M, Islami F, Roshandel G, Khoshnia M, Badakhshan A, Moradi A, Malekzadeh R (2016). Esophageal cancer in Golestan Province, Iran: a review of genetic susceptibility and environmental risk factors. Middle East J Dig Dis.

[5] Zhang HZ, Jin GF, Shen HB (2012). Epidemiologic differences in esophageal cancer between Asian and western populations. Chin J Cancer.

[6] Syrjänen KJ (1982). Histological changes identical to those of condylomatous lesions found in esophageal squamous cell carcinomas. Arch Geschwulstforsch.

[7] Dong HC, Cui XB, Wang LH, Li M, Shen YY, Zhu JB, Li CF, Hu JM, Li SG, Yang L (2015). Type-specific detection of human papillomaviruses in Kazakh esophageal squamous cell carcinoma by genotyping both E6 and L1 genes with MALDI-TOF mass spectrometry. Int J Clin Exp Pathol.

[8] Türkay DÖ, Vural Ç, Sayan M, Gürbüz Y (2016). Detection of human papillomavirus in esophageal and gastroesophageal junction tumors: a retrospective study by real-time polymerase chain reaction in an instutional experience from Turkey and review of literature. Pathol Res Pract.

[9] Ludmir EB, Stephens SJ, Palta M, Willett CG, Czito BG (2015). Human papillomavirus tumor infection in esophageal squamous cell carcinoma. J Gastrointest Oncol.

[10] Georgantis G, Syrakos T, Agorastos T, Miliaras S, Gagalis A, Tsoulfas G, Spanos K, Marakis G (2015). Detection of human papillomavirus DNA in esophageal carcinoma in Greece. World J Gastroenterol.

[11] Liu HY, Zhou SL, Ku JW, Zhang DY, Li B, Han XN, Fan ZM, Cui JL, Lin HL, Guo, et al. Prevalence of human papillomavirus infection in esophageal and cervical cancers in the high incidence area for the two diseases from 2007 to 2009 in Linzhou of Henan Province, northern China. Arch Virol. 2014;159:1393–401. 10.1007/s00705-013-1943-9 24385156

[12] Prakash Saxena PU, Fernandes DJ, Vidyasagar MS, Singh A, Sharan K (2016). Detection of human papilloma virus in patients with squamous cell carcinoma of the esophagusplanned for definitive chemo-radiotherapy, and a study of their clinical characteristics. J Cancer Res Ther.

[13] Pantham G, Ganesan S, Einstadter D, Jin G, Weinberg A, Fass R (2017). Assessment of the incidence of squamous cell papilloma of the esophagus and the presence of high-risk human papilloma virus. Dis Esophagus.

[14] Mehryar MM, Li SY, Liu HW, Li F, Zhang F, Zhou YB, Zeng Y, Li JT (2015). Revalence of human papillomavirus in esophageal carcinoma in Tangshan, China. World J Gastroenterol.

[15] Zhang K, Li JT, Li SY, Zhu LH, Zhou L, Zeng Y (2011). Integration of human papillomavirus 18 DNA in esophageal carcinoma 109 cells. World J Gastroenterol.

[16] Karlsen F, Kalantari M, Jenkins A, Pettersen E, Kristensen G, Holm R, Johansson B, Hagmar B (1996). Use of multiple PCR sets for optimal detection of human papillomavirus. J Clin Microbiol.

[17] Klaes R, Woerner SM, Ridder R, Wentzensen N, Duerst M, Schneider A, Lotz B, Melsheimer P, von Knebel Doeberitz M (1999). Dectection of high-risk cervical intraepithelial neoplasia and cervical cancer by amplification of transcripts derived from integrated papillomavirus oncogenes. Cancer Res.

[18] Hillemanns P, Wang XL (2006). Integration of HPV16 and HPV18 DNA in vulvar intraepithelial neoplasia. Gynecol Oncol.

[19] Klimov E, Vinokourova S, Moisjak E, Rakhmanaliev E, Kobseva V, Laimins L, Kisseljov F, Sulimova G (2002). Human papilloma viruses and cervical tumours: mapping of integration sites and analysis of adjacent cellular sequences. BMC Cancer.

[20] Lee DC, Cheung CY, Law AH, Mok CK, Peiris M, Lau AS (2005). p38 Mitogen- activated protein Kinase-dependent Hyperinduction of tumor necrosis factor alpha expression in response to avian influenza virus H5N1. J Virol.

[21] Yuan B, Li XY, Zhu T, Yuan L, Hu JP, Chen J, Gao W, Ren WZ (2015). Antibody study in canine distemper virus nucleocapsid protein gene-immunized mice. Genet Mol Res.

[22] Arbyn M, Tommasino M, Depuydt C, Dillner J (2014). Are 20 human papillomavirus types causing cervical cancer?. J Pathol.

[23] Doorbar J, Egawa N, Griffin H, Kranjec C, Murakami I (2015). Human papillomavirus molecular biology and disease association. Rev Med Virol.

[24] Gao GF, Roth MJ, Wei WQ, Abnet CC, Chen F, Lu N, Zhao FH, Li XQ, Wang GQ, Taylor PR, Pan QJ, Chen W, Dawsey SM, Qiao YL (2006). No association between HPV infection and the neoplastic progression of esophageal squamous cell carcinoma: result from a cross-sectional study in a high-risk region of China. Int J Cancer.

[25] Kamangar F, Qiao YL, Schiller JT, Dawsey SM, Fears T, Sun XD, Abnet CC, Zhao P, Taylor PR, Mark SD (2006). Human papillomavirus serology and the risk of esophageal and gastric cancers: results from a cohort in a high-risk region in China. Int J Cancer.

[26] Mehryar MM, Li SY, Liu HW, Li F, Zhang F, Zhou YB, Zeng Y, Li JT (2015). Prevalence of human papillomavirus in esophageal carcinoma in Tangshan, China. World J Gastroenterol.

[27] Wells SI, Aronow BJ, Wise TM, Williams SS, Couget JA, Howley PM (2003). Transcriptome signature of irreversible senescence in human papillomavirus-positive cervical cancer cells. Proc Natl Acad Sci U S A.

[28] Bergner S, Halec G, Schmitt M, Aubin F, Alonso A, Auvinen E (2016). Individual and complementary effects of human Papillomavirus Oncogenes on epithelial cell proliferation and differentiation. Cells Tissues Organs.

[29] Ekalaksananan T, Jungpol W, Prasitthimay C, Wongjampa W, Kongyingyoes B, Pientong C (2014). Polymorphisms and functional analysis of the intact human papillomavirus16 e2 gene. Asian Pac J Cancer Prev.

[30] Scheffner M, Romanczuk H, Münger K, Huibregtse JM, Mietz JA, Howley PM (1994). Functions of human papillomavirus proteins. Curr Top Microbiol Immunol.

